# Risk assessment of parasites in Norwegian drinking water: opportunities and challenges

**DOI:** 10.1016/j.fawpar.2021.e00112

**Published:** 2021-01-24

**Authors:** Lucy J. Robertson, Solveig Jore, Vidar Lund, Danica Grahek-Ogden

**Affiliations:** a Parasitology, Department of Paraclinical Science, Faculty of Veterinary Medicine, Norwegian University of Life Sciences, Adamstuen Campus, Ullevålsveien 72, 0454 Oslo, Norway; b Division of Infection Control and Environmental Health, Norwegian Institute of Public Health, Oslo, Norway; c Norwegian Scientific Committee for Food and Environment, Oslo, Norway

**Keywords:** *Cryptosporidium*, Drinking water, *Giardia*, Norway, Risk assessment

## Abstract

Despite the relative prosperity of Scandinavian countries, contamination of the drinking water supply with parasites has occurred on various occasions in the last few decades. These events have resulted in outbreaks of disease involving several thousand cases and/or the necessity for implementation of boil-water advisories. Against this background, in 2008, and again in 2019, the Norwegian Food Safety Authority requested a risk assessment from an independent scientific body regarding parasites in Norwegian drinking water. On each occasion, it was requested that specific questions were addressed.

For the first assessment, data, both of general relevance and specific for Norway, were collected from appropriate sources, as available. Based on some of this information, a quantitative probability model was established and run to estimate the number of cases of waterborne cryptosporidiosis and giardiasis that may be expected in Norway, both in the general public and the immunocompromised, and under conditions where water treatment should be optimal, and also when water treatment efficacy may be compromised by weather conditions.

For the second assessment, approximately a decade after the first, an update on the previous assessment was requested. Differences in information availability and other changes between the two assessments were described; although more data were available at the second assessment, considerable gaps still remained. For both assessments, data on the occurrence of these parasites in the Norwegian population, particularly those infected in Norway, were considered a challenge. However, due to changes in reporting requirements in 2020, the situation was improved for the second assessment. In addition, data were lacking for both assessments on whether animals or humans are most likely to contaminate water sources, and the species and genotypes of these parasites in Norwegian animals. It was also noted that some of the newer data on parasite numbers detected in water samples should be treated with caution. Due to this, further modelling was not conducted. The relevance of risk-based sampling rather than ad hoc sampling of water sources was also addressed.

Despite the data gaps, this article provides an overview of the opportunities provided by conducting such assessments. In addition, some of the challenges encountered in attempting to estimate the risk posed from parasite contamination of water sources in Norway, particularly under predicted conditions of climate change, are described.

## Highlights

• Norwegian drinking water has been contaminated with *Cryptosporidium* and *Giardia.*
• Risk was assessed in both 2008–2009 and 2019–2020 by an independent body.
• A model approach was used in the first assessment and data gaps identified.
• In the second assessment, more data were available, but data gaps remained.
• Main challenges were lack of reliable data on infection and contamination.

## 1 Introduction

Although contamination of drinking water with infectious pathogens is usually associated with poorer countries, wealthy countries also experience problems. Norway, which the World Bank ranked as the richest in the world in terms of wealth per capita in 2018 (Lange et al., 2018), is among those countries for which there have been several occasions where contamination of the drinking water supply has resulted in outbreaks of disease or the necessity for implementation of boil-water advisories. It should be noted that the majority of drinking water in Norway is supplied from surface water sources (lakes and rivers).

In 2004, contamination of the drinking water supply in Bergen, Norway resulted in an outbreak of giardiasis. Over 1500 cases were diagnosed and probably around 6000 persons were infected (Nygård et al., 2006; Robertson et al., 2006). In 2007, detection of contamination of the post-treatment drinking water supply in Oslo, the capital city of Norway, with both bacteria and small quantities of protozoan parasites, resulted in a boil water advisory for 5-days (Robertson et al., 2009). More recently, contamination of the water supply of the Norwegian island municipality of Askøy in 2019 resulted in a waterborne outbreak of campylobacteriosis with over 1500 cases (Hyllestad et al., 2020). One conclusion of this latest waterborne disease outbreak was the importance of water-safety planning, updating infrastructure, and mitigating the likelihood of such outbreaks by risk-based surveillance (Hyllestad et al., 2020). Neighbouring countries of Norway have also experienced waterborne outbreaks of disease, most notably two large outbreaks of waterborne cryptosporidiosis that occurred in Sweden during 2010 and 2011 (Widerström et al., 2014; Bjelkmar et al., 2017), together resulting in over 45,500 cases of cryptosporidiosis.

On the basis of such contamination events and waterborne disease outbreaks, the Norwegian Food Safety Authority (NFSA) has requested the Norwegian Scientific Committee for Food and Environment (VKM) to conduct risk assessments regarding parasites in Norwegian drinking water. The first of these assessments was requested in 2008, and the evaluation published in 2009 (VKM, 2009). In 2019, NFSA requested an update to this assessment from VKM, and this was published in 2020 (VKM, 2020).

The intention with the current article is to provide an overview of the efforts made to assess the risk from parasites, specifically *Cryptosporidium* and *Giardia*, in Norwegian drinking water. In particular, we provide some insights regarding the challenges associated with undertaking such an assessment, and the benefits that can be derived - despite those challenges. It should be noted that the documents from which much of the information provided in this article is based are freely available on the internet (VKM, 2009; VKM, 2020). As both documents are only available in Norwegian, they are of limited accessibility to an international audience.

## 2 Approaches

### 2.1 Mandates for assessment and questions to be addressed

In the first mandate from NFSA to VKM (VKM, 2009) regarding the assessment of the risk from parasites in Norwegian drinking water, 10 specific questions were posed. These were:

1) How important is drinking water, in total or relatively, as a transmission route for cryptosporidiosis or giardiasis in Norway? How might this change in the next few years?
2) How many people in Norway are at risk of becoming ill due to these parasites?
3) Are today's reporting systems adequate? What should be done if necessary?
4) Are humans or animals the most common source of contamination of water in Norway with parasites?
5) How is the risk associated with contamination of the water source compared with contamination of the distribution network?
6) What effect do current water treatment methods have on the removal of these parasites?
7) What monitoring methods are available for these parasites and to what extent are they suitable for waterworks for monitoring purposes?
8) Are models available that owners could use to assess the risk in a specific water supply? If not, is it possible/appropriate to develop such a model for Norwegian water supplies?
9) If current analysis methods are not sufficient or available to meet routine requirements, what should be done, based on an assessment of what is necessary?
10) What general advice can VKM provide to NFSA regarding the risk of parasitic infection via food and drink? If NFSA wants to advise the public on this subject, what should such advice entail and how should it be communicated.

In the second mandate from NFSA to VKM (VKM, 2020), NFSA stated the requirement for updated information on *Cryptosporidium* and *Giardia* in Norwegian drinking water with the goal that owners of waterworks should be able to make the best possible plans for sampling, and that drinking water inspectors should be able to assess these sampling plans and determine whether the samples and analyses necessary are included. As part of the mandate, NFSA included three specific points to be addressed:

1) An update of information on the occurrence of *Giardia* and *Cryptosporidium* in both water sources and treated drinking water in Norway.
2) Information on factors that make it more likely that parasites can be a challenge for the production of sufficient, safe drinking water (for example, the type of raw water source or the activities in the vicinity of the water supply).
3) Criteria that should be met by methods used for analysis of water in order to be adequate, and an update on information regarding the available methods for analysis of drinking water for contamination with *Giardia* and *Cryptosporidium*.

### 2.2 Approaches used

For the first mandate (VKM, 2009), a non-systematic but targeted literature review approach was used to identify information that could be used to answer the specific questions posed by NFSA. Some of this information was available via scientific articles in the published literature. In addition, specific information on, for example, legislation in Norway regarding drinking water supplies and the drinking water treatments in use at that time at Norwegian waterworks, the incidence of reported human cases of giardiasis and cryptosporidiosis, results of analyses of water samples for parasites and the efficiency of the analytical methods used, and tap water consumption patterns of the Norwegian population, was also obtained.

In addition, a quantitative probability model was established using the Norwegian data available, and with awareness of the uncertainty associated with detection of the parasites, variations over time and space, and distribution of parasites within the water mass. The model was made in Microsoft Excel with @Risk as an add-in (Version 4.3.3. – Professional edition, Palisade Corporation 2002). The model was run with 30,000 iterations and with Hypercube sampling and random seed. Details of the model are available online in Appendix II of the risk assessment report (VKM, 2009). In the model, the quantities of parasites (*Cryptosporidium* and *Giardia*) to which the population of Norway may be exposed, and the risk associated with such exposures, were based on five sets of data.

1) The occurrence of parasites in Norwegian water under normal conditions.
2) The effects of water treatment on the parasites in terms of removal or inactivation.
3) The consumption of drinking water by the Norwegian population.
4) Dose-response in both immunocompetent and immunocompromised.
5) Morbidity.

In running this model, two situations were considered:

1) When the water has been treated by a method such as ultraviolet radiation or membrane filtration that is approved as a barrier against parasites and considered to give at least a 2-log reduction in infective parasites under optimal conditions.
2) In other situations, when untreated water or water that is treated by methods that are less effective, is consumed.

In addition, the effects of a situation with excessive precipitation were considered, as this can result in reduced efficiency of water treatment methods that are otherwise considered to be effective (at least 2-log reduction in infective parasites). However, extraordinary events, such as contamination of drinking water with sewage, were not taken into consideration.

Dose-response data included in the model were derived from the international literature, based on the exponential model of Haas et al. (1999) and took into account extreme values that indicate that the probability of exposure to *Cryptosporidium* will result in infection in the general population can vary by a factor of up to 200 (Teunis et al., 2002). For *Giardia*, in contrast, the dose response data were from Teunis and Havelaar (2002) and assumed no variation. In considering the Norwegian population, data on the proportion that were immunocompromised was obtained from the Norwegian Public Health Institute, the Norwegian Prescription Database, and Drug Consumption data. This included, at that time, around 7500 individuals with IgA deficiency, around 3000 individuals infected with human immunodeficiency virus, and around 28,000 individuals treated with immunosuppressants. It was assumed, on the basis of Norwegian food consumption data from 2006, that around 85% (80–90%) of the general population of Norway drank tap water without boiling, and that of those who were immunosuppressed between 10 and 85% drank unboiled tap water (given the advice that is provided to these patients).

In addition, assumptions were made within the model that were of a “worst case scenario” nature. These included: (1) that all *Cryptosporidium* and *Giardia* that have been identified in samples of Norwegian water are infectious to people (are viable, infectious parasites of a species and genotype that can infect humans), and (2) that all exposures were always for the first time (as the effects of previous infection are unclear, and although some immunity develops, the extent of cross-immunity between different infecting species and genotypes has not been fully determined). In addition, it was assumed that among the immunosuppressed all exposures to parasites would result in the establishment of infection.

For the second mandate (VKM, 2020), a primary literature search was conducted using the Advanced Search Builder in the PubMed database (see supplementary material 1).

Further information regarding Norwegian laws and regulations for drinking water, treatments in place in Norwegian water treatment plants, status of the water distribution network, and data on the occurrence of both parasites in water based on analyses were also obtained. This information was compared with data available when the 2009 assessment was conducted to determine whether there was sufficient new information to develop the assessment model further or to re-establish and run the model again with updated information.

For both mandates, there were extensive knowledge gaps and considerable uncertainty was associated with the data available. Both the knowledge gaps and the uncertainty around the available data were considered in the assessments.

## 3 Results and discussion

### 3.1 First mandate: model-based results

Exposure data were based on results from analysis of 475 water samples from 166 different raw water sources over a 6-year period, with analysis using standard methods (ISO, 2006; US EPA, 2012) to obtain occurrence information. Of these samples, the majority were negative for both parasites (392 for *Cryptosporidium* and 415 for *Giardia*); the positive samples contained between 1 and 3 cysts or oocysts. Whereas 6 samples were found to contain 3 *Cryptosporidium* oocysts, only 1 sample was found to contain 3 *Giardia* cysts. The recovery efficiency of the analytical method used was based on the results of 15 spiking studies. At that time, the waterworks register at the Norwegian Public Health Institute indicated that around half of the Norwegian population were supplied with drinking water treated by UV disinfection (Table 1).

**Table 1:** Drinking water treatments among the population of Norway in 2009

Treatment process	Number of people supplied^a^	Proportion of population supplied (%)
Coagulation/filtration only^b^	935,000	19
Membrane filtration	141,000	3
UV treatment only^b^	1,399,000	29
Coagulation/filtration and UV^b^	995,000	21
Other (including groundwater supply)	1,269,300	26

a Estimated population of Norway at the time of data collection (2009): 4,799,300.

b People supplied by water with UV treatment in total was 2,394,000 and by coagulation and filtration in total is 1,930,000, of whom 995,000 are supplied with water with both treatments.

The log-reduction effects of the different water treatments were derived from published literature (Ongerth and Pecoraro, 1995; Ormerod and Lund, 2004), as summarised in Table 2.

**Table 2:** Estimated log reductions in infectious *Cryptosporidium* and *Giardia* in drinking water supplies in Norway based on treatments in place and precipitation quantity.

Water treatment process	*Cryptosporidium*: approx. Log reduction	*Giardia*: approx. Log reduction
Coagulation/filtration (optimal)	2.7–3.1	3.1–3.6
Coagulation/filtration (during excess precipitation)	1.5	1.3
Membrane filtration	3–6	3–6
UV treatment (alone, optimal)	2–6	2–6
UV treatment (alone, during excess precipitation)	1.5–4	1.5–4
UV treatment (following filtration, during excess precipitation)	2–5	2–5
Coagulation/filtration and UV	Sum of individual values	
Other (including groundwater supply)	0	0

Based on these data, the Norwegian population water consumption patterns, and known immune status, the daily individual probability for infection with *Cryptosporidium* or *Giardia* from drinking tap water was calculated to range from 5 × 10^−6^ to 8 × 10^−4^ for *Cryptosporidium* and 0 to 4 × 10^−4^ for *Giardia,* depending on immune status and whether there was excess precipitation (Table 3).

**Table 3:** Mean daily individual probability for infection from drinking contaminated tap water in Norway in 2009.

		Treated drinking water	
		Optimal treatment	Excess precipitation
*Cryptosporidium*	General public	5 × 10^−6^	3 × 10^−5^
	Immunocompromised	2 × 10^−5^	8 × 10^−4^
*Giardia*	General public	7 × 10^−7^	1 × 10^−5^
	Immunocompromised	0	45 × 10^−4^

These data can be extrapolated to estimate the expected number of cases of cryptosporidiosis and giardiasis per day from contaminated water based on these probabilities of exposure (Table 4).

**Table 4:** Expected daily number of cases of cryptosporidiosis and giardiasis from drinking contaminated tap water in Norway in 2009.

		Treated drinking water	
		Optimal treatment	Excess precipitation
Cryptosporidiosis	General public	5	40
	Immunocompromised	1	18
Giardiasis	General public	1	15
	Immunocompromised	0	10

### 3.2 First mandate: answers to the questions

In addition to the model-based results, the first assessment (VKM, 2009) provided answers to the questions posed (see Section 2.1). The individual answers provided at that time are summarised in the Supplementary Material, Table 1.

In brief, the answers showed that data that could be used to determine the relative importance of the waterborne route of infection was lacking for both parasites, particularly as cryptosporidiosis was seldom diagnosed and, at that time, not notifiable (except in AIDS diagnoses). Furthermore, although most notified cases of giardiasis had been infected abroad, samples from people presenting at their doctor with diarrhoea, but who had not been abroad, were usually not analysed for parasites. This presumably resulted in considerable underdiagnosis of giardiasis among people who had been infected while in Norway. Nevertheless, it was suggested that despite the large waterborne outbreak of giardiasis in 2004, the lack of other outbreaks or cases associated with water indicated that Norwegian drinking water was probably a minor transmission vehicle for *Giardia*. Data were also considered to be lacking regarding whether humans or animals were most likely to contaminate water sources, and, given that both parasites may be zoonotic, was suggested to probably vary between water sources, depending on the catchment area. Furthermore, the transmission of parasites via drinking water was described as being likely to reduce in the coming years due to the greater implementation of water treatments that are effective against parasites. However, it was also noted that climate changes, specifically milder and wetter winters, may contribute to greater survival and higher risk of contamination of water sources. Although it was difficult to compare the likelihood of infection from contaminated water sources compared with from contamination within the distribution network, lack of integrity within the distribution network was mentioned as a problem.

Methods were, in general, available at that time for analysis of water samples for contamination with these parasites, but monitoring illness in the community and ensuring water treatment processes were operating as intended are important for early detection of outbreaks and for ensuring water safety. It is also important to be aware of occasions when parasite removal/inactivation is compromised or suboptimal. Parasite analysis of water samples was noted to be especially important for risk mapping of water sources and for investigating outbreaks or cases in the community. The importance of water providers having knowledge of relevant water catchments and potential contamination sources was also emphasised.

The conclusions from this first mandate (VKM, 2009) were that the incidence of cryptosporidiosis and giardiasis the Norwegian population is probably relatively low, although under-estimated. In addition, the data suggest that there is widespread, but low-level, contamination of water sources. This is reflected in the lack of outbreaks, which may also reflect that many water treatment works have efficacious treatments (e.g., UV or membrane filtration) against parasites in place. In order to have better information on cryptosporidiosis in the Norwegian population, mandated reporting was recommended. The probabilistic model indicated that, within the confines of the wide uncertainties, most consumers are not exposed to parasites in their drinking water on a daily basis. Water monitoring for parasite contamination was considered to be particularly useful for assessing individual catchments and for outbreak investigation, but the analyses should be conducted by an accredited laboratory; at that time, none of the laboratories in Norway were accredited for these analyses.

### 3.3 Second mandate; changes from 2009

The second assessment (VKM, 2020) was considered an update on the first, with the intention of describing any changes in the assumptions on which conclusions in the first mandate were based. Although some information and data had increased only minimally from 2009, such as information about infections in animals, additional information or data expansion were available on other aspects. Furthermore, whereas improvements or developments had been implemented in some aspects of relevance (Table 5), for others, such as new methods for analysing water for parasites or replacement of pipes in the distribution network, had either not occurred or was minimal; leakage in the distribution network had decreased only marginally from 29.9% in 2009 to 28% in 2018.

**Table 5:** Changes in information, data, and activities regarding *Cryptosporidium* and *Giardia* in Norway since 2009

Area of changes	2009	2020
Diagnosis of human infection	Diagnostics generally performed by microscopy or antigen testing. Few medical microbiology laboratories analyse for parasites and both training and experience is lacking. No reference laboratory.	Several laboratories use molecular methods for diagnosis of parasitic infections. These may be of higher sensitivity than older methods and also the algorithm for testing may be broader as many pathogens can be tested simultaneously in a multiplex format. A reference laboratory is being established.
Reporting of human infection	Only giardiasis reportable.	Cryptosporidiosis also reportable as from 2012. Numbers of cases have risen for both infections from around 2016 (see Table 2, supplementary data).
Water treatment and distribution.	Around 50% of the population receive drinking water that has been treated with UV.	Around 85% of the population receive drinking water that has been treated with UV. Although there seem to be more ozonisation in water treatment plants, other effective methodologies (such as membrane filtration) has not expanded so much. Most large waterworks have upgraded their treatments to be effective against parasite contamination.
Water analyses	No accredited laboratories and few water suppliers request analysis of water samples.	One laboratory in Norway accredited for analysis of water for these parasites, however several laboratories offer this service without being accredited. Around 870 samples of raw water and 230 samples of treated drinking water were analysed between 2009 and 2018. Although most samples were negative, some positive reports are recorded, generally with low numbers of parasites. See also text.

Other information accrued since 2019 regarded further outbreaks of cryptosporidiosis and giardiasis in Norway. However, none of these were shown to be definitively associated with water contamination, but largely associated with animal contact (Rimšelienė et al., 2011; Lange et al., 2014), contact with infected people (Johansen et al., 2015), or of unknown source (Rimšelienė et al., 2011). One further outbreak of cryptosporidiosis was associated with contaminated self-pressed apple juice (Robertson et al., 2019) and water was not implicated in this outbreak either. Despite this additional information and the changes from 2009 listed in Table 5, data that indicated further refinement of the model or repeat running of the model were not apparent. Indeed, among the water analysis data provided via NFSA, some notable reports, such as one 10 L sample of raw water containing 14 oocysts and another 10 L raw water sample containing 41 oocysts, did not seem to have been followed up; no information was available regarding measures that were implemented regarding these unusually high counts of parasites or whether there had been any infections identified in the community served by that water source. Similarly, a 10 L sample of treated drinking water was reported to contain 4 *Cryptosporidium* oocysts, again without any further information. Information was also lacking regarding whether molecular analysis had been conducted to determine species in these instances.

Despite the recommendation from 2009 that only accredited laboratories or laboratories providing documented ring-test results (participating regularly in proficiency tests as part of an external quality assurance scheme) should be used for analysing water samples, this recommendation has apparently not been followed. Thus, the unexpected results of high contamination in some raw water samples or oocysts in treated drinking water samples, with no further information, should therefore be considered with caution. Furthermore, these questionable data suggested that repeated running of the model developed in the previous mandate (VKM, 2009) would not provide more useful information, and actually could result in less accurate outputs due to the uncertainty, particularly around the high-count data.

### 3.4 Second mandate: answers to the questions

In addition to comparing the status of both information and data with that available for the first assessment (VKM, 2009), the second mandate provided answers to three additional questions (see Section 2.1). The responses are summarised below.

#### 3.4.1 Updated information on the occurrence of *Giardia* and *Cryptosporidium* in Norwegian drinking water (water sources and treated water)

Although further information had been accrued on the occurrence of *Giardia* and *Cryptosporidium* in both water sources and treated drinking water in Norway, the circumstances for the analysis were unknown. Thus, it could not be determined whether the sampling was based on a risk-sampling strategy or not, nor whether the analyses had been conducted by an accredited laboratory; in several cases this did not seem to be the case. Therefore, knowledge on the occurrence of these parasites in Norwegian water sources seems to be no clearer than over a decade previously; where remarkable results were reported, information on whether this was associated with a particular event was lacking and any actions following this finding, including molecular analysis to determine species and/or genotype, were not provided.

#### 3.4.2 Information on factors that make it more likely that parasites can be a challenge for the production of sufficient, safe drinking water

Although the literature review indicated that climate change could affect contamination of drinking waters sources with protozoan parasites due to run-off from the environment to surface waters, which are the primary sources of drinking water in Norway, whether this occurs in Norway has apparently not been investigated. A pilot study investigating contamination of irrigation water with various microbes noted that *Cryptosporidium* and *Giardia* were not detected in water samples collected at the study sites during the dry/warm period, but samples collected after rainfall contained both parasites (Paruch et al., 2015). Targeted sampling of drinking water sources could provide relevant information, but this has not apparently been conducted.

It was additionally noted that climate change may also affect water colour and quality (humus content), which, in turn, could affect the efficacy of some drinking-water treatment methods (Health Canada, 2019). For example, it is has been well established for many years (e.g., Hall and Packham, 1965) that natural organic matter (NOM) in water exerts a coagulant demand that interferes with reducing turbidity and *Cryptosporidium* breakthrough has been reported at a water treatment plant due to an increase in colour in source water (James et al., 2016). In addition, NOM exerts a chemical oxidant demand that needs to be met before necessary pathogen log-inactivation levels can be achieved and studies have also shown that the presence of humic acid particles significantly affects UV disinfection efficiency (Health Canada, 2019). Transmittance of UV of wavelength 254 nm is affected by dissolved and particulate matter that reduces its penetration through water and, in general, a 50% reduction in dose results from every 10% decrease in UV transmittance of the water (Health Canada, 2019).

#### 3.4.3 Criteria that should be met by methods used for analysis of water in order to be adequate, and an update on information regarding the available methods of analysis of drinking water for contamination with *Giardia* and *Cryptosporidium*

The standard methods (ISO, 2006; US EPA, 2012) used worldwide are also used in Norway for analysis of water for contamination with *Cryptosporidium* and *Giardia*. These methods have not been substantially altered since publication. The recovery efficiency of the method varies according to water quality and other factors, but, in Norway, in low-turbidity samples, this is often around 60%. In the one laboratory in Norway that is accredited for these analyses, recovery efficiencies over 30% are necessary to be considered acceptable. One relevant problem of the method is that any contamination in a reservoir is likely to be distributed unevenly (a Poisson distribution); given the relatively low volume of water analysed compared with the volume of the reservoir, false negative results may be obtained. It is therefore recommended that sampling should be risk-based; the sampling point for a given water source should be selected with care and that sampling occasions should also be targeted towards the question to be addressed.

### 3.5 Overall outcomes of the assessments: opportunities and challenges

NFSA's request to obtain an assessment of the risk from contamination of drinking water with *Cryptosporidium* and/or *Giardia* is without doubt well intentioned and understandable, based on the outbreaks of waterborne giardiasis and waterborne cryptosporidiosis that have occurred in Norway and in neighbouring countries. The initial mandate resulted in the presentation of relevant information on basic questions and attempted to use these data, along with other necessary data from the international literature, within a modelling approach to estimate cases of waterborne giardiasis and cryptosporidiosis. Places where data were absent were also pinpointed. These gaps included the lack of information on the occurrence of these parasites in the Norwegian population, particularly those who had been infected in Norway; an absence of information on whether animals or humans were most likely to contaminate Norwegian water sources; limited information on genotypes and species of parasites in different Norwegian animals; an absence of information on the effects of different weather conditions on the likelihood for contamination. In addition to these Norway-specific information gaps, more general information was also found to be lacking, such as the duration of immunity following exposure, and the extent of cross-immunity between species or genotypes.

Within the decade between mandates, more relevant data had become available due to improved diagnostics, a potentially broader algorithm for investigating patient samples for parasites, and cryptosporidiosis being a notifiable infection. In addition, more water samples have been analysed for contamination with *Cryptosporidium* and *Giardia*. The latter, however, represents a challenge, as not all laboratories that conduct these analyses are accredited to do so, and information on unexpected results (such as detection of *Cryptosporidium* in treated water or high quantities of parasites in raw water) is lacking, including the basis for the analysis, any follow-up investigation, and any resultant disease in the community. Furthermore, many of the data gaps identified in 2009 remain, including the extent of infection in the human population, whether animal faeces or human sewage are the predominant source of water contamination, and the effects of weather conditions (heavy rain, snow melt) on water contamination.

As with the previous assessment (VKM, 2009), the more recent assessment (VKM, 2020) was an opportunity to urge water providers to consider their water supply catchments, routines for disinfection, and potential for contamination to occur. Should analysis of samples be considered a useful step to obtain more information, a systematic sampling plan based on the specific characteristics of each source and watershed, along with specification of the questions to be addressed, should be implemented. This should be a collaborative process between those with on-the-ground knowledge of the supply and catchment, and those with experience in risk-based sampling plans.

## Declaration of Competing Interest

The authors declare that they have no known competing financial interests or personal relationships that could have appeared to influence the work reported in this paper.

## Appendix A Supplementary data

Supplementary material Image 1
