# Supplementary material for: Risk assessment of parasites in Norwegian drinking water: opportunities and challenges
Source: Food Waterborne Parasitol. 2021 Jan 24;22:e00112. doi: 10.1016/j.fawpar.2021.e00112 (PMC7930114; doi:10.1016/j.fawpar.2021.e00112)
Supplement: Supplementary file 1 — Supplementary material [file mmc1.docx]

Supplementary Material for:

**Risk assessment of parasites in Norwegian drinking water: opportunities and challenges**

**Lucy J. Robertson*, Solveig Jore, Vidar Lund, Danica Grahek-Ogden**

1. Primary literature search information (VKM, 2020)

The primary literature seach was conducted using the Advanced Search Builder in the PubMed database ([www.ncbi.nlm.nih.gov/pubmed](http://www.ncbi.nlm.nih.gov/pubmed) ) in January 2020 with the keywords (((((giard*[Title/Abstract]) OR cryptosporid*[Title/Abstract]) OR kryptosporid**[Title/Abstract]) AND water[Title/Abstract]) AND ( "2009/01/01"[PDat] : "3000/12/31"[PDat] )). All keywords were searched in the Title / Abstract field. Two of the authors scanned titles and abstracts of the 1210 articles identified for their relevance to the mandate.

**Reference**

VKM [Norwegian Scientific Committee for Food and Environment] (2020). *Giardia* og *Cryptosporidium* i norsk drikkevann [*Giardia* and *Cryptosporidium* in Norwegian drinking water]. VKM Report 2020:07. ISBN: 978-82-8259-345-8. Available at: <https://vkm.no/risikovurderinger/risikovurderinger.4.59777ce315d3abb2351cb0ad.html?y=2020&s=publisert&g=hygiene-og-smittestoffer>

**2.** **First mandate: summary of answers to the questions provided in assessment**

| **Questions** | **Responses provided (VKM, 2009)** |
| --- | --- |
| 1. How important is drinking water, in total or relatively, as a transmission route for cryptosporidiosis or giardiasis in Norway? How might this change in the next few years? | Cryptosporidiosis is rarely diagnosed in Norway and therefore it is difficult to say which routes of infection are most important. There has been only one known major outbreak of waterborne giardiasis, and none of waterborne cryptosporidiosis. Minor outbreaks / individual cases may not have been identified. Although most drinking water in Norway comes from surface water sources, as only one major disease outbreak from parasites in raw water has occurred, drinking water may be an unimportant source for these diseases. However, as samples from people who have not been abroad are seldom analysed, the incidence of domestic infection is therefore probably strongly underdiagnosed.  Installation of equipment in waterworks to remove and/or inactivate parasites will reduce the risk of waterborne transmission. In the next few years, most large waterworks will have UV disinfection, and some will also have coagulation.  Climate change with increased rainfall could result in increased contamination of drinking water sources with parasites and milder winters could prolong their survival in the environment. |
| 1. How many people in Norway are at risk of becoming ill due to these parasites? | Generally, everyone who does not have acquired immunity to these parasites is susceptible to infection. However, not everyone infected will necessarily develop symptoms. Outbreaks that have occurred in Norway suggest that a relatively large proportion are likely to develop symptoms of disease. People who are regularly exposed to small quantities of parasites are likely to develop some immunity, but exposure to a high dose or new genotype / species may still result in development of disease. It is unknown how many people in Norway have immunity to these parasites.  Although immunocompromised people are at greater risk of developing disease, immunocompetent people can also become ill. Although we estimate that 38,000 may be at increased risk due to compromised immunity, susceptibility will vary within the group. |
| 1. Are today's reporting systems adequate? What should be done if necessary? | Currently giardiasis is notifiable to the reporting system, but cryptosporidiosis is only notifiable  as an AIDS-defining diagnosis. However, a notification requirement for cryptosporidiosis has been recommended. When this is implemented, all cryptosporidiosis and giardiasis cases diagnosed  will be reported. However, an earlier survey has found that few microbiology laboratories in Norway perform these analyses, samples from those who have not been abroad are rarely analysed for parasites, and there are also issues with the diagnostic tests used. Thus, limitations in diagnostics mean that  the number of patients in Norway is probably underestimated. Improved diagnostics and the notification requirement are likely to improve the data reported. |
| 1. Are humans or animals the most common source of contamination of water in Norway with parasites? | We currently do not have the data to answer this question. Wastewater (human sewage) contains high  numbers of both parasites, and both parasites are widespread in wild and domestic animals in Norway.  However, not all animals are infected with species or genotypes infectious to people. For example,  *Giardia* cysts are often found in sheep samples, but these genotypes are rarely found in samples from  people. As different water sources are exposed to different contamination pressures the likelihood varies from waterworks to waterworks. Catchment assessment and monitoring could provide useful data. Molecular analysis of *Cryptosporidium* oocysts isolated from people in Norway could provide information regarding relevant animal species as sources of infection (*C. hominis*, for example, rarely infects animals), but will provide no information on whether the vehicle of infection was water or something else. |
| 5) How is the risk associated with contamination of the water source compared with contamination of the distribution network? | Water treatments that inactivate or remove parasites should reduce contamination of drinking water coming from the source. Water contamination in the distribution network can occur when there is  negative pressure in the pipe and external water is contaminated, or contaminated water is pumped in at excess pressure. As usually only smaller volumes of water, reaching fewer consumers, are contaminated in this way, sporadic cases rather than outbreaks are more likely to occur. Finding the infection source for sporadic cases is difficult, and therefore we lack reliable information on the incidence of disease associated with contamination of water in the distribution network. There is considerable leakage in the Norwegian water distribution network, and if repairs/replacements are not conducted these are likely to increase and may result in an increased risk of contamination. |
| 1. What effect do current water treatment methods have on the removal of these parasites? | Removal and/or inactivation of these parasites using currently available water treatments can result in hygienically safe water supply from the source water. Treatment methods used in Norway that can act as appropriate hygienic barriers against parasites include UV disinfection, membrane filtration, and coagulation (chemical precipitation). These treatments should be effective, even during heavy rainfall. |
| 1. What monitoring methods are available for these parasites and to what extent are they suitable for waterworks for monitoring purposes? | Monitoring can be divided into indirect methods (monitoring disease in consumers or monitoring water treatment processes or indicators) or direct methods (water sample analysis to detect parasites). Effective disease monitoring enables trend following and outbreak detection. However, today's monitoring systems would mean that outbreaks are only discovered after the peak has passed. Monitoring disease in the community provides useful information but does not necessarily indicate waterborne transmission as infection may occur via other routes. Monitoring that processes in the waterworks are operating correctly is important as these are barriers against contamination of the drinking water network. This could include particle monitoring of coagulation systems and membrane filter systems, and UV sensor readings.  Analysing water samples for parasites is useful for determining potential associations with contamination and identifying catchment risks. Water sample analysis is also important for investigating outbreaks or cases potentially associated with water. In such instances, samples should be analysed as early as possible. |
| 1. Are models available that could owners could use to assess the risk in a specific water supply? If not, is it possible/appropriate to develop such a model for Norwegian water supplies? | Various models have been developed, but few have been subsequently checked for whether outcome predictions are supported by reality. This is due to various factors including: diagnostic issues, varying pathogenicities, multiple potential infection sources and routes, lack of knowledge about geographical and temporal variations in infection pressure, and the difficulty in predicting unexpected events. Establishing such models for Norway is possible and this may provide waterworks with guidelines based on available knowledge. Qualitative models are already available based on relevant conditions in water sources, catchments, and treatment processes, as well as the size of the waterworks. Some models suggest monitoring requirements and have been used in Bergen and Trondheim. Quantitative models may indicate barrier requirements within water treatment and may attempt to estimate the risk associated with detection of parasites in water, in order to determine needs for further action. Such models usually have high uncertainty due to data input deficits but can be useful to assess effects of various measures, estimate risk limits, and calculate whether safety improves following repeated analyses. Due to lack of validation of such models, their appropriateness must be constantly evaluated. Regardless of model, waterworks with vulnerable water sources should have knowledge of establishments and activities found in the catchment area, and the extent to which these could result in contamination of the water supply. |
| 1. If current analysis methods are not sufficient or available to meet routine requirements, what should be done, based on an assessment of what is necessary? | There are currently no medical microbiology laboratories in Norway with a reference function for *Giardia* and *Cryptosporidium* diagnostics. Such analyses are undertaken relatively rarely, and training in the techniques is limited. Implementation of diagnostic techniques for human infection is recommended; animal samples are regularly analysed for these parasites and the methodology could be transferred.  Although current techniques for analysing water for these parasites are expensive and sub-optimal, there has been considerable progress in the past 20-30 years. Use of FITC-labelled monoclonal antibodies and  fluorescence microscopy remains the gold standard. Molecular typing methods are important for determining genotype in outbreaks or when water sources are contaminated. Current methods for the detection of parasites require training, particularly when microscopy is the detection method; *Cryptosporidium*, which is only 3-5 µm in diameter is particularly difficult. In Norway, three or four laboratories have established routines for detecting parasites in water samples, but not all participate in ring tests, and none are accredited (although one, at the veterinary school, is working towards this). |
| 1. What general advice can VKM provide to NFSA regarding the risk of parasitic infection via food and drink? If NFSA wants to advise the public on this subject, what should such advice entail and how should it be communicated? | This assessment considers only drinking water; infection via contaminated food or other infection routes has not been assessed. Both parasites are common in Norway, and protection of drinking water sources against contamination varies throughout the country. People who are regularly exposed to the parasites may develop immunity. Advice on the risk to the public should be coordinated with the Norwegian Institute of Public Health. |

**Reference**

VKM [Norwegian Scientific Committee for Food and Environment] (2009). Risikovurdering av parasitter i norsk drikkevann [Risk assessment of parasites in Norwegian drinking water]. VKM Report 2009:28. ISBN: 978-82-8082-342-7. Available at: <https://vkm.no/risikovurderinger/risikovurderinger.4.59777ce315d3abb2351cb0ad.html?g=hygiene-og-smittestoffer&s=publisert&y=2009>
